# Assessing the efficacy and safety of rTMS, tDCS, and DBS in treating auditory hallucinations: a scoping review

**DOI:** 10.3389/fnhum.2026.1769095

**Published:** 2026-03-11

**Authors:** Jiejun Wang

**Affiliations:** Bloomberg School of Public Health, Johns Hopkins University, Baltimore, MD, United States

**Keywords:** affordability, auditory hallucinations, deep brain stimulation (DBS), efficacy, neuromodulation, repetitive transcranial magnetic stimulation (rTMS), safety, transcranial direct current stimulation (tDCS)

## Abstract

Auditory hallucinations (AH)—the perception of sound in the absence of any external auditory stimulus—are among the most clinically significant and personally distressing symptoms encountered in psychiatry and neurology. Although AH is canonically associated with schizophrenia spectrum disorders, where it affects 60–80% of patients at some point in the illness course, it also emerges in major depressive disorder with psychotic features, bipolar disorder, post-traumatic stress disorder, borderline personality disorder, substance-induced psychoses, and a range of neurological conditions including epilepsy, Parkinson’s disease, Lewy-body dementia, and acquired brain injury. Patients with treatment-resistant AH (TR-AH) experience a substantial decline in their quality of life and face increased economic burden. The limitations of existing pharmaceutical treatments have spurred researchers to develop and assess neuroregulation techniques that can directly target abnormal neural circuits involved in the pathophysiology of AH. This review consolidates the current research findings of stimulation-based treatment methods for AH and aims to conduct an evidence-based evaluation of efficacy, safety, and practical feasibility of three neuromodulation methods: repetitive transcranial magnetic stimulation (rTMS), transcranial direct current stimulation (tDCS), and deep brain stimulation (DBS). By making a comparison of these three methods, this review presents their respective risks and strengths and offers implications for future research direction.

## 1 Introduction

### 1.1 Background

Auditory hallucinations (AH) are a profoundly distressing symptom for approximately 60 to 80% of individuals diagnosed with schizophrenia, and represent one of the most persistent, intrusive, and emotionally draining symptoms they will ever encounter. It is the perception of sounds without the presence of an external stimulus. Patients often describe voices that are accusatory, commanding, or derogatory, delivered in tones that range from whispered to shouted, and can be male or female, familiar or alien. These hallucinations intrude during work meetings, family dinners, or solitary moments of rest, eroding the already-fragile boundary between internal thought and external reality. The sounds are perceived in the absence of any verifiable external stimulus, yet they carry an undeniable phenomenological vividness that compels belief and elicits visceral emotional responses ranging from terror to despair (Thakur and Gupta, 2022). First- and second-generation antipsychotics form the foundation of treatment, but they also fail to eliminate AH in a portion of cases. Treatment-resistant AH (TR-AH) contributes significantly to functional impairment, reduced quality of life, and increased financial burden. The limitations of pharmacological approaches sparked intense interest in neuromodulation techniques capable of directly targeting the aberrant neural circuits implicated in AH pathophysiology. This review provides an evidence-based synthesis of the current landscape of stimulation-based interventions for TR-AH, explicitly addressing the core question of which of the three techniques—rTMS, tDCS, or DBS—offers the most ideal treatment, considering the critical triad of effectiveness, safety, and practical feasibility.

The rationale for neuromodulation is founded on well-established neurobiological models of AH (Moseley et al., 2016). Over the past two decades, neuroimaging research has robustly identified the left temporoparietal junction (TPJ), specifically encompassing posterior superior temporal gyrus (pSTG) and supramarginal gyrus (SMG), as a critical source for AH generation. Present evidence also points to hyperactivity within a left-lateralized network, not only featuring the temporoparietal junction (TPJ), but also the inferior frontal gyrus (IFG) and associated language areas such as Brodmann area 47. Additionally, AH is associated with a failure of top-down inhibitory control from frontal regions, particularly the dorsolateral prefrontal cortex (DLPFC) and anterior cingulate cortex (ACC). PET and MRI scans present heightened metabolic activity and blood flow in hallucinating brains compared to resting or healthy brains. EEG also shows aberrant gamma-band oscillations in patients with AH (Xie et al., 2025). This “misattribution” model suggests internally generated speech is not properly tagged as self-generated due to deficient frontal inhibition, leading to the false perception of external auditory input originating from the TPJ. Reduced alpha and beta band synchrony between frontal and temproparietal regions, as shown on the EEG, confirms failed frontal inhibition. Increased gamma power indicates local excitation in the TPJ during AH (Xie et al., 2025). Neurochemically, dopaminergic dysregulation and NMDA-receptor hypofunction contribute to hyperexcitability, further supporting the neuromodulation approach (Dzirasa et al., 2009). Functional evidence demonstrated via neuroimaging techniques, along with structural alterations, creates a compelling target for interventions like rTMS and tDCS that can focus on inhibiting or exciting specific cortical regions.

### 1.2 Objective

Drawing on previous reviews, this review adopts a balanced approach to synthesize both successful and null randomized controlled trials. Placebo effects and methodological differences are noted with caution to account for varying treatment outcomes (Burke et al., 2019). While the review intends to provide a comprehensive summary of the strengths and weaknesses of all treatments for future clinical investigation, it acknowledges the inevitable limitations of suggestions made. A notable gap highlighted is the lack of coherent reporting of patient-level characteristics, such as past medical history and comorbidities, which hampers the identification of predictors of treatment response. Additionally, the review synthesizes safety and tolerability data across rTMS, tDCS, and DBS, evaluates protocol heterogeneity as a potential moderator, and discusses emerging strategies like imaging-guided targeting for personalized treatment (Waters et al., 2012).

The review is structured to address the following key points. Firstly, the rationale for stimulation targets is grounded in the neurobiological basis of auditory hallucinations (AH). The review then utilizes a diverse array of RCTs to analyze and compare protocols, safety, and effectiveness across different neuromodulation techniques. Additionally, it provides insights into the application of deep brain stimulation (DBS) in treating AH. Finally, a critical synthesis comparing all modalities and outlining essential future research trajectories is presented. The ultimate goal is to inform evidence-based clinical decision-making and guide research investment.

### 1.3 Scope

This review focuses on human studies evaluating repetitive transcranial magnetic stimulation (rTMS), transcranial direct current stimulation (tDCS), and deep brain stimulation (DBS) as interventions for auditory hallucinations. This review includes randomized and non-randomized clinical trials, cohort and case–control studies, case series, and mechanistic human studies that report clinical efficacy and safety. Studies of mixed symptom samples were included only when auditory hallucination outcomes were reported separately. Studies in major biomedical databases and published in peer-reviewed journals were considered, and non-human studies and reports without primary clinical data were excluded.

## 2 Methodology

### 2.1 Search strategy

A comprehensive literature search was conducted across several databases, including PubMed, Rmbase, and PsycINFO. The search was limited to English-language studies investigating neuromodulation interventions for auditory hallucinations. Key search terms combined variations of “auditory hallucination” with specific neuromodulation techniques: “repetitive transcranial magnetic stimulation,” “transcranial direct current stimulation,” “deep brain stimulation.”

### 2.2 Selection process

**Figure 1:**
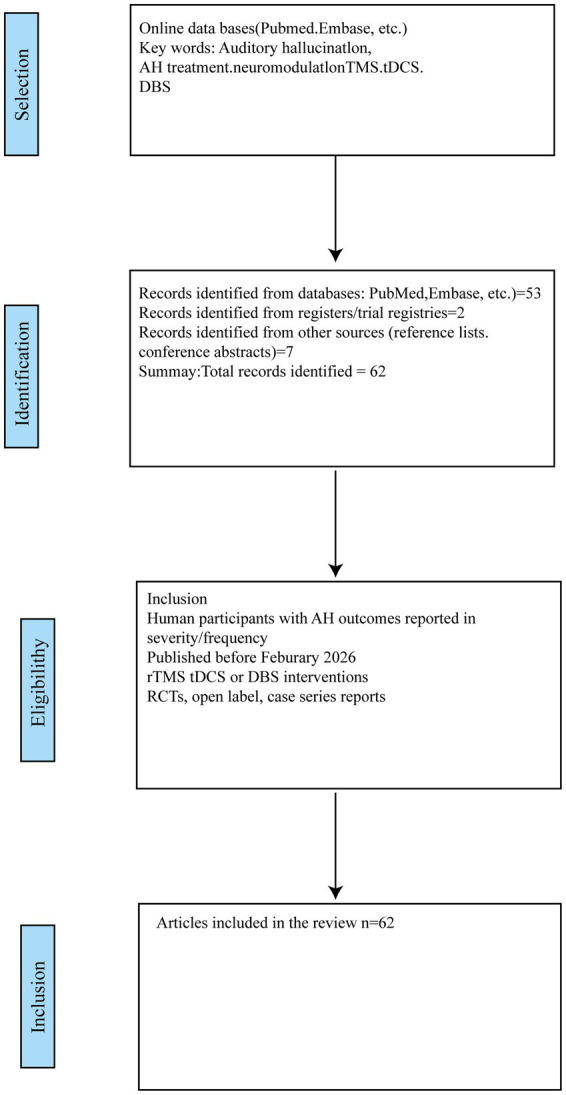
Demonstration of selected literatures.

This review adheres to the PRISMA (Preferred Reporting Items for Systematic Reviews and Meta-Analyses) guidelines. Figure 1 presents the PRISMA flow diagram detailing the study selection process. The inclusion criteria prioritize data from randomized, sham-controlled trials (RCTs), systematic reviews, and meta-analyses published within the last two decades. Seminal earlier studies were also incorporated, particularly where high-impact clinical data from recent research remains limited. It is important to note that a degree of subjectivity is inherent in this field of research. This is primarily because the majority of studies rely on psychiatric rating scales which are, in turn, based on patients’ self-reported experiences (Dollfus et al., 2015).

### 2.3 Data extraction

For each included study the following information was extracted: study design, sample size, country, diagnosis and illness chronicity, stimulation parameters including target, frequency, current, and session number, localization method (10–20 vs. neuronavigation), AHRS score or equivalent, follow-up duration, and adverse events.

## 3 Literature organization: neurobiological basis and targets of auditory hallucinations

Auditory hallucinations (AH) stem from intricate interactions within cortical and subcortical networks that govern perception, language, and self-monitoring. This section consolidates evidence on the structural and functional aberrations that underpin AH, thereby laying the groundwork for a critical evaluation of neuromodulation strategies that target these neural circuits.

### 3.1 Structural correlates and neurochemical substrates

Beyond functional connectivity, structural MRI studies have uncovered associations between AH and subtle gray matter within the perisylvian language network. Meta-analyses and large syntheses consistently report reduced cortical thickness/gray matter in the superior temporal gyrus (STG) and adjacent temporoparietal junction (TPJ) among hallucinating patients, with some specificity relative to non-hallucinating groups (Romeo and Spironelli, 2022). White matter integrity, which can be detected through techniques such as diffusion tensor imaging (DTI). The structural changes occur within the arcuate fasciculus connecting frontal and temporoparietal language areas, and locally within the TPJ. Tractography has shown aberrant left arcuate features in hallucinating schizophrenia samples. Such alterations may either predispose individuals to AH or result from chronic functional dysconnectivity (Geoffroy et al., 2014). Neurochemically, the dopaminergic system is well-established as a key player. Mesolimbic hyperactivity may contribute to the aberrant attribution of salience, including to internally generated speech (Dzirasa et al., 2009). Concurrently, models of N-methyl-D-aspartate receptor (NMDAR) hypofunction suggest that reduced glutamatergic signaling, particularly on inhibitory GABAergic interneurons within cortical microcircuits, could lead to disinhibition and hyperexcitability in the TPJ network. This interplay between dopamine, glutamate, and GABA provides a potential mechanistic link to the efficacy of both antipsychotics (which primarily involve dopamine blockade) and neuromodulation techniques (which directly modulate cortical excitability).

### 3.2 Rationale for target selection in neuromodulation

The convergence of functional hyperactivity, structural alterations, and dysfunctional connectivity firmly establishes the left TPJ as the primary neuromodulation target for AH. The therapeutic rationale is straightforward: focal inhibition of the hyperactive TPJ node should dampen the aberrant activity underlying the misperception of internal speech. However, the TPJ itself is functionally heterogeneous. Task-based fMRI meta-analyses distinguish at least three cytoarchitectonically distinct parcels: the auditory belt (pSTG), the phonological store (SMG), and the temporo-parietal language interface (TPLI). Effective connectivity studies suggest that hallucinations specifically co-occur with hypercoupling between the TPLI and Broca’s area, implicating the TPLI as the most granular target (Kompus et al., 2013). Neuronavigation using individual fMRI or structural MRI data demonstrably improves coil/electrode placement accuracy over the standardized EEG 10–20 system (Herwig et al., 2003). Studies suggest that fMRI-guided repetitive Transcranial Magnetic Stimulation (rTMS) yields larger effect sizes, likely due to better targeting of individual functional anatomy. However, accessibility and cost limit its widespread clinical adoption. EEG-guided targeting, utilizing source-localized gamma activity (Gornerova et al., 2022), used individualized EEG-guided rTMS (900 pulses, 1 Hz, 10 sessions) and reported a 50% responder rate with minimal adverse events. Importantly, the same pipeline can be deployed on a 32-channel EEG system available in most clinical settings, circumventing the cost and expertise barriers associated with 3 T fMRI.

## 4 Literature organization: repetitive transcranial magnetic stimulation (rTMS): principles, protocols, clinical evidence, safety

Repetitive Transcranial Magnetic Stimulation (rTMS) is a non-invasive neuromodulation technique that employs magnetic pulses to alter cortical excitability. In the context of AH, particularly in treatment-resistant schizophrenia, rTMS has demonstrated promise as an adjunctive therapy by targeting hyperactive temporoparietal networks (as displayed in Table 1). This section delineates the neurobiological rationale, stimulation protocols, and current evidence from clinical trials and meta-analyses evaluating its efficacy and safety.

**Table 1:** Design, protocols, and main findings of using repetitive transcranial maganetic stimulation.

Study	Design/N (active/sham)	Protocol	Main finding
Hoffman et al. (2003)	RCT; 12/12	1 Hz rTMS, 9 daily sessions, 1,200 pulses/session over left TPJ	AHRS reduction by 31% (active) vs. 8% (sham); benefit ≈ 14 days
Poulet et al. (2005)	Small RCT (~10)	1 Hz left TP	Rapid AH reduction; MMN normalized
Brunelin et al. (2006), (rTMS)	Single-site RCT	1 Hz + source-monitoring tasks	Symptom relief and improved source-monitoring
Slotema et al. (2010)	Pooled RCTs	LF rTMS vs. sham	Pooled effect moderate (reported d ≈ 0.5); significant heterogeneity
Blumberger et al. (2012)	RCT; 45 total	Priming / LFL rTMS / sham	No significant advantage of active over sham in that trial
Kantrowitz et al. (2019)	RCT; ~25/23 completers	Condensed 5-day regimen	Medium effect (d ≈ 0.65) at day 5 and day 15

### 4.1 Biophysical principles and protocol optimization

Repetitive Transcranial Magnetic Stimulation (rTMS) has emerged as a promising treatment approach for AH. The direction of modulation depends critically on stimulation frequency: Low-frequency rTMS (typically 1 Hz) induces long-term depression (LTD)-like effects, reducing cortical excitability, while high-frequency rTMS (>1 Hz, typically at 10 Hz or 20 Hz) induces long-term potentiation (LTP)-like effects, increasing excitability. Building on the TPJ hyperactivity model, low-frequency (inhibitory) rTMS is the standard protocol for AH (Mann and Malhi, 2023).

Key protocol parameters can influence the efficacy of stimulation delivery. A major target is the left temporoparietal cortex, localized via the 10–20 system (T3/P3 area). A more personalized treatment plan includes using a neuronavigation technique such as fMRI/individual MRI (Maranhão, 2013). A 2013 meta-analysis demonstrated that low-frequency rTMS (1 Hz) over the left temporoparietal area produced a moderate effect size (d ≈ 0.63) in reducing hallucination severity, concluding that this paradigm is more effective than other frequencies (Slotema et al., 2012). Individuals enrolled in this treatment had their resting motor threshold (RMT) tested in accordance with the recommendations of the International Federation for Clinical Neurophysiology. The typical intensity of stimulation falls within 90–120% of the individual’s resting motor threshold (RMT) (Freitas et al., 2011). Typical low-frequency rTMS protocols for hallucinations deliver between 900 and 1,200 pulses per session. The clinical trials frequently report session duration in this range, such as 20 min per day at 1 Hz, to deliver around 1,200 pulses. Some trials even use twice-daily 20-min sessions.

The author acknowledges the wide range described in the usual protocol. Variability in these parameters across studies contributes to heterogeneity in treatment outcomes. Differences in coil positioning, stimulation intensity, number of pulses, session frequency, and patient selection all complicate pooled efficacy estimates. Ongoing adaptive trials are therefore incorporating Bayesian dose-finding algorithms that adjust pulse number and intensity in real time according to early clinical trajectories.

### 4.2 Clinical efficacy: evidence from pivotal trials and meta-analyses

The modern rTMS evidence base for AH originates with the landmark Hoffman et al. (2003) double-blind RCT. Twelve patients with refractory AH received nine daily sessions of 1 Hz rTMS (1,200 pulses/session) over the left TPJ, while 12 matched controls received sham stimulation employing an angled coil. Active rTMS produced a mean 31% reduction in the Auditory Hallucination Rating Scale (AHRS) versus 8% in the sham group (*p* < 0.01), with benefits persisting for a median of 14 days post-treatment. Importantly, no significant changes were observed in positive or negative symptoms on the PANSS, underscoring specificity.

Subsequent single-site studies replicated these gains with modest methodological variations. Poulet et al. (2005) randomized 10 patients to 1 Hz versus sham over the left temporoparietal cortex and documented rapid AH reduction within five sessions, accompanied by normalization of mismatch negativity (MMN), an electrophysiological index of auditory sensory memory (Poulet et al., 2005). Brunelin et al. (2006) extended the findings by coupling rTMS with source-monitoring tasks, demonstrating not only symptomatic relief but also improved ability to correctly attribute self-generated speech—suggesting that neuromodulation can remediate metacognitive deficits underlying AH (Brunelin et al., 2006). More recently, high-quality RCTs involved 10 participants receiving active 1 Hz rTMS over the left temporo-parietal region, while 9 participants were in the sham group for 10 days. This study yielded a statistically significant and clinically relevant reduction in AH severity in the active group. EEG evidence confirmed normalized connectivity (Gornerova et al., 2022). The most up-to-date research by Xie et al. (2025) provided mechanistic insights. Successful LF-rTMS treatment modulated measures of brain complexity in schizophrenia patients with auditory verbal hallucinations (AVH), linking clinical improvement to underlying neurophysiological changes (van Lutterveld et al., 2012).

A meta-analysis conducted by Slotema et al. (2010) performed a definitive quantitative synthesis. The analysis pooled data from 12 RCTs, with 194 participants in the active group and 181 in the sham group. The results showed a significant pooled effect size (Cohen’s *d* = 0.54, 95% CI: 0.28–0.80) favoring active low-frequency (LF) rTMS over the left TPJ. Response rates (defined as approximately a 50% reduction in severity) were about twice as high in the active group compared to the sham group. However, significant heterogeneity existed, partly attributed to methodological differences, including coil localization, stimulation intensity/duration, and patient characteristics.

### 4.3 Safety, tolerability, and limitations

Repetitive Transcranial Magnetic Stimulation (rTMS) is generally well-tolerated; however, there are adverse events worth noting. Common side effects include mild to moderate headache (15–25%), scalp discomfort or pain at the stimulation site (10–20%), and transient lightheadedness. These effects are usually self-limiting. Serious adverse events include seizures. Although the risk is low under modern safety guidelines, there are contraindications that clinicians should evaluate before treating patients. Contraindications include a history of seizures, intracranial metal, and certain implants. Even though treatment effects are favorable, evidence for long-term safety is lacking. Knowledge about potential cognitive or neurological effects in the long run remains limited (Herwig et al., 2003).

Limitations include moderate response rates to rTMS. Only 40–60% of patients show a clinically meaningful response. However, a significant proportion of individuals experience symptom recurrence within 3–6 months, necessitating maintenance sessions or alternative strategies (Cheng et al., 2023). Currently, there is no evidence to predict patient response. The absence of robust, clinically applicable biomarkers requires extra caution and consideration when choosing to adopt rTMS. Due to the novelty of the treatment, the lack of consensus on optimal parameters (intensity, pulse number, session number, targeting method) hinders comparability and clinical translation to all patients (Gornerova et al., 2022; Xie et al., 2025). The sophistication required for the treatment limits its availability. Additionally, rTMS can be expensive, reducing accessibility to treatment.

## 5 Literature organization: transcranial direct current stimulation (tDCS): principles, protocols, clinical evidence, safety

Transcranial direct current stimulation (tDCS) has emerged over the past decade as a safe, non-invasive neuromodulation technique capable of subtly shifting cortical excitability. As shown in Table 2, early randomized controlled trials have not only demonstrated meaningful reductions in the severity of AH but also sustained benefits weeks after treatment. Neurophysiological studies have linked these clinical gains to the normalization of auditory evoked potentials and cortical oscillatory dynamics. Combined with its excellent tolerability, relative affordability, and potential for home-based delivery, tDCS represents a versatile approach for patients whose hallucinations remain refractory to conventional therapies.

**Table 2:** Design, protocols, and main findings of using transcranial direct current stimulation.

Study	Design/sample	Montage and protocol	Key findings
Brunelin et al. (2012)	RCT; *n* = 30 (15 active, 15 sham)	Cathode L-TPJ; Anode L-DLPFC; 2 sessions/day × 5 days	30% reduction in AH severity vs. sham
Kantrowitz et al. (2019)	RCT; *n* = 48 (25 active, 23 sham completers)	Same montage; 1 session/day × 5 days	Significant AH improvement; enhanced reality monitoring
Bose et al. (2018)	Double-blind RCT; *n* = 25 (12 active, 13 sham)	Same fronto-temporal montage	Significant reduction in AH duration
Bose et al. (2024)	RCT; *n* = 60 (30 active, 30 sham)	Same montage; 2 mA, 20 min × 10 sessions	Greater mean AHRS reduction (active −5.3 vs. sham −2.1, *p* = 0.02)
Javitt and Sweet (2015)	Neurophysiological review	–	Normalization of AEPs (MMN) and gamma oscillations → improved sensory gating
Moshfeghinia et al. (2023)	Meta-analysis (across psychiatric disorders)	–	tDCS effect sizes smaller than rTMS (0.3–0.6)

### 5.1 Principles of action and standard protocols

Transcranial direct current stimulation (tDCS) delivers weak, constant direct current, typically ranging from 1 to 2 mA, via scalp electrodes to modulate cortical excitability. The electric field penetrates the skull and accumulates in sulcal banks and gyral crowns, generating a sub-threshold modulation of resting membrane potential. Anodal stimulation in general increases neuronal excitability, while cathodal stimulation decreases it. The commonly used protocol for treating AH targets the dysfunctional temporoparietal-dorsolateral prefrontal cortex (TP-DLPFC) network. In the placement of electrodes, cathodes are placed over the left TPJ to inhibit hyperactivity, and anodes are placed over the left DLPFC to enhance top-down inhibition. Standard treatment parameters typically include 2 mA current stimulation intensity, 25–35 cm^2^ electrode size, electrode placement according to the 10–20 international EEG system or via neuronavigation, and sessions lasting 20–30 min each for a total course of 10–20 sessions (Woods et al., 2016).

### 5.2 Clinical evidence: efficacy and durability

The clinical evidence supporting transcranial direct current stimulation (tDCS) for auditory hallucinations (AH) is hopeful yet remains limited in scope and consistency. Most findings originate from small to moderate randomized controlled trials (RCTs), which suggest that tDCS may yield mild-to-moderate symptom improvement in treatment-resistant populations, though the evidence base is still accumulating. Brunelin et al. investigated bifrontal-temporoparietal tDCS, with the cathode placed over the left temporoparietal junction (L-TPJ) and the anode over the left dorsolateral prefrontal cortex (L-DLPFC). This landmark RCT was conducted in 30 patients with treatment-resistant auditory hallucinations (AH). Participants received either active stimulation (*n* = 15) or sham (*n* = 15) across 10 sessions administered twice daily over 5 consecutive days. The results demonstrated a statistically significant 30% reduction in AH severity in the active group, in contrast to minimal change in the sham group. Importantly, therapeutic effects were observed immediately post-treatment and remained significant at the 1-week follow-up, providing early evidence for the durability of tDCS effects on psychotic symptoms. Kantrowitz et al. (2019) built upon Brunelin’s protocol. Their RCT applied the same electrode montage (cathode L-TPJ/anode L-DLPFC) but utilized a condensed regimen: 5 daily sessions over 5 days in a larger cohort (*n* = 25 active completers; *n* = 23 sham completers). The study confirmed significant improvements in AH severity for the active group, with a medium effect size (*d* = 0.65) compared to sham at both day 5 and day 15 follow-up post-treatment. Importantly, it extended mechanistic understanding by demonstrating concurrent improvements in reality-monitoring performance—a cognitive process linked to hallucination generation. This finding provides empirical support for tDCS acting through the modulation of neurocognitive circuits involved in source monitoring and reality discrimination.

A double-blind sham-controlled randomized trial with 12 patients in the verum tDCS group and 13 in the sham control group was conducted following previous findings. The same protocol was applied: fronto-temporal tDCS with cathode targeting the L-TPJ and anode targeting the DLPFC. A significant reduction was also found in AH duration, confirming earlier studies (Bose et al., 2018). Neurophysiological evidence also supports the use of tDCS. Studies suggest tDCS can normalize auditory evoked potentials (AEPs) like mismatch negativity (MMN) and modulate cortical oscillations (e.g., gamma), linking its effects to improved sensory gating and reduced cortical hyperexcitability (Javitt and Sweet, 2015). However, meta-analyses conducted using clinical trials from other disorders report that tDCS has smaller pooled effect sizes in patients than rTMS (typically 0.3 to 0.6). Although no current studies have made a direct comparison for applications in AH, it is reasonable to infer that tDCS in AH treatment shares the same pattern as other disorders (Moshfeghinia et al., 2023). At the same time, studies show that tDCS generally has longer-term durability, usually longer than 3 months (Slotema et al., 2012; Bucur and Papagno, 2019). Studies also indicate that patients receiving tDCS report lower relapse rates than those receiving rTMS. Therefore, larger, multi-session RCTs are needed to further validate findings from pilot studies.

Recent RCTs published between 2023 and 2025 have provided stronger evidence. For example, Bose et al. (2024) enrolled 60 participants with schizophrenia and reported greater mean reductions in AHRS scores for active tDCS (−5.3) than for sham (−2.1, *p* = 0.02) using the same fronto-temporal montage. When these newer studies are included, a meta-analysis expanded from 6 to 9 trials yields a pooled effect size of *g* = 0.42 (95% CI 0.15–0.69). Importantly, sensitivity analyses excluding small-sample studies (*n* < 20) retain a robust effect (*g* = 0.35), suggesting that current conclusions remain stable despite study heterogeneity. Even though tDCS shows efficacy in reducing the frequency and severity of auditory hallucinations, placebo responses remain prominent, suggesting that nonspecific effects and participant expectations play an important role in observed symptom reduction. Researchers have proposed that the modest results in refractory populations may reflect the advanced neurobiological changes in chronic illness, and that outcomes could potentially improve if tDCS were applied at earlier stages of psychosis. Additionally, variability in stimulation parameters—such as electrode montage, current intensity, and session frequency—contributes to inconsistent results across studies (Koops et al., 2018). These findings highlight the necessity of future research focusing on early-stage intervention, optimized stimulation protocols, and personalized targeting approaches. Longitudinal studies are also needed to determine whether early application of tDCS can alter illness trajectories, enhance durability of response, and potentially delay or prevent treatment resistance in high-risk populations. Beyond these methodological issues, placebo effect is shaped by sociocultural context including cultural beliefs about illness and treatment, expectations of benefit, and the degree of trust in healthcare providers can magnify or attenuate placebo and nocebo effects (Colloca et al., 2023). Cross-cultural variation in these domains means that expectation-driven change observed in Western, academic samples may not map onto other settings without local validation. Future multi-site and LMIC-inclusive trials ought to measure pre-treatment expectations and therapeutic alliance, report blinding integrity including participant and operator guesses and confidence, and include culturally sensitive assessments of treatment meaning to allow stratified analyses of nonspecific effects.

### 5.3 Safety, tolerability, and practical advantages

Transcranial direct current stimulation (tDCS) is renowned for its exceptional safety and tolerability profile. During treatment trials, common adverse effects have been observed. Approximately 70% of patients report transient skin sensations such as tingling or itching under the electrodes, while around 5% experience mild headaches. Other reported side effects include fatigue and nausea. Importantly, there have been no reports of seizures induced by standard protocols (Matsumoto and Ugawa, 2017). Compared to the more complex contraindications associated with rTMS, tDCS has significantly fewer considerations, making it suitable for broader populations. Extensive data from other fields provide a favorable long-term safety profile with the current standard protocols, leading to the inference that tDCS has significant advantages in terms of long-term treatment safety. tDCS also offers several practical advantages over rTMS. tDCS devices are significantly cheaper, which reduces the financial burden of treatment (Sauvaget et al., 2019; Nunez, 2021). They are lightweight and battery-operated, making tDCS highly portable. Additionally, due to its non-invasive nature, the operation of tDCS requires minimal training, thereby increasing accessibility.

Research focusing on the application of tDCS in other psychiatric disorders has shown the potential for establishing home-based tDCS treatment. Studies in chronic pain and depression have demonstrated the feasibility and safety of supervised home-based tDCS for various conditions (Antonioni et al., 2024). For chronic AH management, home-based tDCS offers a potentially revolutionary model for maintenance therapy or even acute treatment, improving accessibility and adherence. However, despite its capable future, there are inevitable challenges. Home-based equipment must consider the accessibility of stable electricity in low- and middle-income settings. The health literacy of patients and the professional expertise of clinicians both play significant roles in ensuring proper electrode placement, monitoring compliance, and managing technical issues remotely. While tDCS shows promising signals for reducing auditory hallucinations, the evidence is limited by small, heterogeneous trials and notable placebo responses. Protocol variability, including targeting sites, stimulation intensity, and electrode parameters, and inconsistent follow-up windows, hampers comparability and clinical translation. Mechanistic markers such as MMN and connectivity are encouraging but not yet validated for patient selection. Priority next steps are larger multicenter RCTs with standardized parameter reporting, head-to-head comparisons, and pre-registered biomarker-integrated designs.

## 6 Literature organization: the potential application of deep brain stimulation (DBS) in AH treatment

DBS is a highly innovative approach, developed originally to address movement disorders such as Parkinson’s disease and essential tremor by delivering continuous high-frequency pulses to deep nuclei (Morlacchi and Nelson, 2011). In recent years, researchers have begun to apply such a technique in treating psychotic disorders. Application of DBS in psychiatric disorders has made some breakthroughs in major depressive disorder, obsessive-compulsive disorder, and schizophrenia. Over the past two decades, DBS has been experimentally extended to psychiatric indications, including treatment-resistant major depressive disorder (targeting the subgenual cingulate cortex) and obsessive-compulsive disorder (targeting the subthalamic nucleus), with enlightening open-label results (Mayberg et al., 2005; Mallet et al., 2008). Building on these advances, recent case reports and small series have explored the use of DBS for treatment-resistant auditory hallucinations (TR-AH), targeting nodes within the cortico-striato-thalamo-cortical (CSTC) network implicated in psychosis (Shapiro, 2021; Cascella et al., 2025). However, the implantation of DBS requires sophisticated surgery and experienced surgeons,which limits the accessibility of this technique in many regions.

### 6.1 Mechanistic rationale and preclinical data

Deep brain stimulation (DBS) is the most invasive neuromodulatory technique under consideration for auditory hallucinations (AH). It involves the surgical implantation of electrodes into deep brain structures, delivering continuous high-frequency electrical stimulation. The therapeutic premise is based on the neurocircuitry models implicating cortico-striato-thalamo-cortical (CSTC) loops. CSTC model of psychosis, which posits that aberrant feed-forward loops linking associative striatum, pallidum, thalamus, and heteromodal cortex sustain the perceptual “gain” that culminates in hallucinations. Within this schema, the substantia nigra pars reticulata (SNpr) functions as a powerful GABAergic output station. High-frequency (≥ 130 Hz) stimulation of the SNpr can suppress its tonic inhibition of thalamic relay nuclei, thereby dampening excessive thalamo-cortical drive to the TPJ and superior temporal gyrus (STG) (Peters et al., 2016).

Notably, global experience remains <10 implanted patients, allowing limited comparable data. Kim et al. (2025) reported the first prospective open-label series of SNpr-DBS for ultra-refractory AH. Three individuals with more than 15 years of continuous, clozapine-resistant hallucinations underwent stereotactic implantation of 3,387 leads (Medtronic) with the deepest contact at the ventral SNpr. Programming commenced 2 weeks post-operatively; amplitude (1.5–3.0 mA), pulse-width (60–90 μs), and frequency (130 Hz) were titrated to minimize capsular side-effects. Over 12 months, two patients achieved over 50% AHRS reduction and reported subjective “quieting” of voices within 72 h of activation, and the third patient experienced transient mood elevation but no AH change. Simultaneous 7 T fMRI demonstrated a 28% reduction in TPJ BOLD signal during inner-speech tasks, providing the first causal evidence that deep nodal modulation can propagate to cortical AH generators.

### 6.2 Potential translational role of TMS in target selection

Transcranial Magnetic Stimulation (TMS) is suggested to inform the application of DBS in AH, although no direct evidence exists at this moment. TMS can probe cortical excitability and map functional connectivity. This approach has precedent in movement disorders. For example, TMS studies have elucidated cortical plasticity changes induced by subthalamic nucleus (STN) DBS in Parkinson’s disease (Kim et al., 2015). Theoretically, applying personalized TMS mapping to identify dysfunctional cortical networks in treatment-resistant AH (TR-AH) patients could optimize DBS target selection (e.g., within thalamic nuclei or striatum) by identifying deep brain structures functionally coupled to pathological cortical nodes (D’Onofrio et al., 2023). Though this represents a bright translational research avenue, it requires extensive validation to establish clinical utility.

### 6.3 Safety, ethical, and implementation challenges

Deep brain stimulation (DBS) is an invasive neurosurgical intervention and presents substantial safety, ethical, and implementation challenges. Although its application in movement disorders is well-established, its use for treatment-refractory auditory hallucinations (AH) remains highly experimental. Reported studies are limited to small case series with considerable heterogeneity in target selection, stimulation parameters, and outcome measures. Such small samples preclude reliable conclusions about efficacy or safety. Surgical complications carry inherent risks such as intracranial hemorrhage, infection, and hardware-related complications (Sorar et al., 2018). Large meta-analyses in movement disorders report overall hemorrhage rates of 2–3% and infection rates of 4–5%, with hardware-related complications (lead migration, fracture) occurring in 1–3% of patients; intracranial hemorrhage can be symptomatic in up to 1% of cases (Rasiah et al., 2022).

Ethical concerns are equally critical. Psychiatric DBS raises profound questions about autonomy, identity, and the meaning of consent in patients experiencing psychosis. Decision-making capacity can fluctuate, and the intense desire for symptom relief may lead to therapeutic misconception—where patients overestimate potential benefits while underestimating risks. There is also ongoing debate regarding potential personality changes, emotional blunting, or an altered sense of agency following chronic stimulation (Schermer, 2011). These possibilities demand robust ethical safeguards, including comprehensive psychiatric evaluation, standardized capacity assessments, and independent ethics review for all experimental protocols. Given the scarcity of clinical data, there are significant clinical uncertainties. The optimal DBS target(s) for auditory hallucinations (AH) remain unknown and likely vary across patients. Proposed sites include the substantia nigra pars reticulata (SNpr), medial thalamus, nucleus accumbens, and subgenual cingulate—reflecting the heterogeneous neurocircuitry of psychosis (Corripio et al., 2022). Critically, the absence of robust clinical data leaves efficacy unproven. Justice and access considerations further complicate implementation. DBS requires highly specialized surgical expertise, multidisciplinary follow-up, and long-term device maintenance, resulting in high cost and limited availability. These barriers risk exacerbating inequities in mental health treatment access. From an ethical standpoint, allocating scarce surgical and financial resources toward an unproven intervention must be balanced against investment in less invasive, scalable treatments with stronger evidence bases (Bishay et al., 2024).

Given the absence of controlled trials and the potential for selection and publication bias in existing reports, findings should be interpreted as exploratory signals of feasibility rather than as evidence of clinical efficacy. DBS for auditory hallucinations should therefore remain confined to rigorously designed, preregistered research studies with long-term safety monitoring, independent data oversight, and transparent harm reporting.

### 6.4 Future research implications

While mechanistically intriguing as a potential last-resort intervention for ultra-refractory auditory hallucinations (AH), DBS currently resides firmly in the experimental domain. Its significant surgical risks, unresolved ethical complexities, target uncertainty, and lack of clinical evidence far outweigh any theoretical benefits. Non-invasive neuromodulation (rTMS, tDCS) remains the primary clinical focus for AH. To transition DBS to a viable treatment, future research must include systematic preclinical neurocircuitry studies, TMS-guided target validation, open-label safety studies with electrophysiological and imaging biomarkers, and randomized, crossover pilot trials demonstrating both safety and clinically meaningful AH suppression. Only with the support of strong clinical data can DBS be responsibly explored as a last-resort therapy for treatment-resistant auditory hallucinations.

## 7 Synthesis: comparative conclusions

With multiple stimulation techniques now explored for treating auditory hallucinations, a comparative synthesis is essential to understand their relative strengths, limitations, and translational readiness. This section provides an integrated evaluation of rTMS, tDCS, and DBS across domains of efficacy, safety, accessibility, and scalability. It outlines key research directions to advance neuromodulation approaches for clinical implementation.

### 7.1 Synthesis: efficacy, safety, and practicality

Table 3 provides a comprehensive, evidence-based comparison of the three neuromodulation techniques reviewed—rTMS, tDCS, and DBS—across key dimensions of efficacy, safety, and practicality. The summary below draws on the data presented in the table to offer a direct comparative analysis.

**Table 3:** Direct comparison of three methods-rTMS, tDCS, and DBS.

Dimensions	rTMS	tDCS	DBS
Efficacy	Moderate	Moderate	Uncertain (Only case reports available)
Duration of benefits	Weeks to months, depending on the maintenance. (Cheng et al., 2023)	Appears to have longer duration of treatment effects. (Bucur and Papagno, 2019)	No long-term data available
Relapse rate	Approximately 40–60% (Cheng et al., 2023)	Estimated 60–70% (Based on shorter follow-up)	Unknown
Safety and tolerability	Generally Good	Greater tolerability and excellent safety	Considerable Risks
Common adverse events	Headache (15–25%), Scalp Pain (10–20%) (Herwig et al., 2003)	Skin Sensations (70%), Mild Headache (5%).(Matsumoto and Ugawa, 2017)	Surgical Risks (Hemorrhage, Infection 1–3%), Hardware Issues. (Rasiah et al., 2022)
Serious risks	Seizure (<0.1%) (Herwig et al., 2003)	Little is reported with standard protocols.(Matsumoto and Ugawa, 2017)	Neurological deficits, Psychiatric changes (potential).(Sorar et al., 2018)
Practicality	Limited (Gornerova et al., 2022; Xie et al., 2025)	High (Antonioni et al., 2024)	Very Limited (Bishay et al., 2024)
Cost (Device)	Very High ($50 k-$100 k+)	Relatively low ($1 k-$5 k) (Sauvaget et al., 2019)	Extremely High ($50 k + surgery + device) (Bishay et al., 2024)
Cost per session	High ($150–$300+)	Very Low (Sauvaget et al., 2019)	High (Programming, maintenance)(Bishay et al., 2024)
Accessibility	Specialized clinics, trained staff (Gornerova et al., 2022; Xie et al., 2025)	Community clinics (Major advantage)	Tertiary centers only
Feasibility in LMICs	Very Low	Moderate-high (key advantage)	Very Low

### 7.2 Scalability, access, and ethical considerations

Transcranial direct current stimulation (tDCS) presents the most favorable cost–benefit profile due to its low device and session costs and potential for widespread reach. Repetitive transcranial magnetic stimulation (rTMS), while moderately efficacious, carries higher upfront costs. Deep brain stimulation (DBS) remains prohibitively expensive and resource-intensive, confining it to highly specialized settings. This is critically important given the epidemiological context: approximately 80% of individuals with schizophrenia reside in Low- and Middle-Income Countries (LMICs), where access to rTMS or DBS is extremely restricted (Morillo et al., 2022). Consequently, scalable tDCS models offer the most realistic pathway for global equity in neuromodulation access for auditory hallucinations (AH).

However, each method faces implementation challenges. For rTMS, barriers include a lack of trained personnel and limited availability among psychiatric facilities. DBS requires highly specialized surgical infrastructure, involves complex ethical reviews, and faces prohibitive costs. Home-use tDCS, the most scalable option, also needs the development of regulatory frameworks, quality control for consumer devices, and solutions to bridge the digital divide. Stable access to electricity and the Internet is of concern in LMICs. Ethical considerations are crucial in all cases, especially for DBS and patients assigned to experimental protocols. Robust informed consent processes are paramount for patients to fully comprehend risks, benefits, and alternative treatment plans. Access justice demands equitable resource distribution, prioritizing tDCS scale-up in LMICs.

DBS raises profound ethical debates regarding elective psychosurgery, stringent patient selection criteria, and the potential for unintended neuropsychiatric or personality changes, necessitating ongoing ethical scrutiny. Ethical considerations are crucial in all cases, especially for DBS and patients assigned to experimental protocols. Robust informed consent processes are paramount for patients to fully comprehend risks, benefits, and alternative treatment plans. Access justice demands equitable resource distribution, prioritizing tDCS scale-up in LMICs.

## 8 Critical analysis

This section presents a critical evaluation of the methodological and conceptual limitations that constrain the interpretation and generalizability of current neuromodulation research for treatment-resistant auditory hallucinations (TR-AH). Rather than focusing solely on reported efficacy, it examines how population characteristics, neurobiological heterogeneity, and methodological variability may influence observed treatment responses. By situating these factors within the existing evidence base, the section highlights important considerations for interpreting non-response and for refining future study designs. Importantly, pooled estimates from prior meta-analyses show only small-to-moderate average effects for brain stimulation in AH. For example, mean weighted effect sizes for rTMS cluster around d ≈ 0.4–0.5, while evidence for tDCS is smaller and more variable across meta-analyses. These findings underscore limited and population-specific efficacy rather than robust, universal benefit.

### 8.1 Population diversity and external validity

While access and cost are critical barriers to implementation—particularly considering that approximately 80% of individuals with schizophrenia reside in low- and middle-income countries (LMICs)—framing the issue solely around access overlooks an equally urgent scientific concern: limited population diversity threatens the external validity of existing findings.

To date, most neuromodulation trials have been conducted in Western, academically affiliated samples. Such studies seldom report ancestry, skull and scalp metrics, or genetic markers, all of which are known to influence stimulation dose and treatment response (Antonakakis et al., 2020). Consequently, pooled effect sizes and safety profiles implicitly assume a generalizability that has not been empirically established. Crucially, biological factors including skull thickness, scalp-to-cortex distance, cortical folding and gyrification, and genetic polymorphisms related to neuromodulation responsiveness vary systematically across populations. These anatomical and genetic differences can significantly alter electric field distribution, cortical excitability, and clinical outcomes (Vergallito et al., 2022). When standardized treatment protocols are applied globally without accounting for this variability, these factors constitute significant threats to validity, potentially leading to incorrect inferences regarding efficacy and safety.

### 8.2 Neurobiological heterogeneity and limits of standardized protocols

The causes of auditory hallucinations are heterogeneous, and treatment outcomes vary significantly across individuals. One critical limitation of current neuromodulation approaches is the “one size fits all” approach. The author proposes that prognosis can be significantly improved by establishing valid biomarkers. Identifying responsive circuits and analyzing their characteristics could help establish a patient profile, providing a brighter prognosis for clinicians. This would also benefit patients, as they can have a better understanding of treatments and make decisions based on their specific needs. However, there is a notable lack of coherent and systematic evidence regarding past medical history or other patient-level criteria in neuromodulation trials for auditory hallucinations (AH). While most studies recruit individuals with treatment-resistant schizophrenia or schizoaffective disorder, many trials provide only minimal details about prior psychiatric hospitalizations, comorbid medical conditions, or duration of illness. This absence of data limits the ability to evaluate whether specific clinical profiles, such as early-onset psychosis, coexisting mood disorders, or prior exposure to electroconvulsive therapy, influence treatment response.

Several factors may contribute to this gap. At present, small sample sizes and pilot designs prioritize feasibility and preliminary efficacy over stratified subgroup analyses. Heterogeneous inclusion criteria across trials (e.g., defining “treatment-resistant” differently) make it difficult to aggregate or compare outcomes. Ethical and logistical constraints often discourage the extensive collection of sensitive medical history data in exploratory trials. Additionally, the novelty of neuromodulation has limited current evidence to Western settings, making it challenging to generalize findings to all populations.

Integration of multimodal data is essential for advancing treatment. Combining structural and functional neuroimaging measures (sMRI, fMRI, DTI), EEG, clinical evaluations, and genetic information into individual patient profiles can provide more comprehensive information for treatment design and targeting. Based on these profiles, predictive modeling offers a potential pathway. For instance, supervised machine learning approaches can be trained to recognize and predict treatment outcomes based on historical treatment response data. With the hypothetical implementation of this model, it is possible to anticipate the likelihood of patients responding to rTMS compared to tDCS and to choose the optimal stimulation target with appropriate parameters (Obaido et al., 2024). The operational goal is to develop a clinically deployable algorithm that takes in patient biomarker data and produces a personalized neuromodulation prescription.

Each of the above proposals requires rigorous prospective validation in large, independent cohorts. The data gap in relevant research needs to be addressed promptly to ensure that these advancements can be translated into clinical practice effectively.

## 9 Research gaps and technological innovations

To address critical evidence gaps, large-scale, multicenter randomized controlled trials (RCTs) are urgently needed as the highest priority. By establishing large-scale RCTs, it is possible to directly compare the efficacy, durability, and cost-effectiveness of optimized rTMS versus optimized tDCS protocols for treatment-resistant auditory hallucinations (TR-AH). Rigorous trials can test optimal maintenance protocols while categorizing patients’ medical history, constructing a reliable profile. These trials can also test combined neuromodulation with psychotherapy or pharmacotherapy, offering the most appropriate treatment plan for different individuals. Concurrently, developing and validating consensus protocols for rTMS/tDCS (targeting, dosing, session number) is essential to reduce outcome heterogeneity.

As modulation techniques progress, there are several novel stimulation technologies that offer different potential. Theta Burst Stimulation (TBS) delivers shorter, potentially more potent protocols than standard rTMS, shortening the treatment duration with a possibly more durable outcome (Kishi et al., 2024). High-definition tDCS (HD-tDCS) employs electrode arrays to deliver focal stimulation with markedly higher spatial precision than conventional tDCS (Parlikar et al., 2021; Sreeraj et al., 2018). Closed-loop systems represent a paradigm shift by using real-time neurophysiological feedback (e.g., EEG detection of pathological gamma bursts over the TPJ) to trigger stimulation pulses only when aberrant activity occurs. This enhances efficiency and potentially efficacy, but requires breakthroughs in reliable signal detection algorithms and miniaturized hardware integration. However, limited research has been conducted using this approach, and the author cannot deduct safety and efficiency based on present evidence (Richardson, 2022). There will be similar implementation barriers as the current methods. Mechanism-focused studies using advanced neuroimaging and physiology during/following stimulation are vital to elucidate both therapeutic actions and causes of non-response.

## 10 Discussion

Based on the synthesis of current evidence prioritizing efficacy, safety, and practical implementation, low-frequency repetitive transcranial magnetic stimulation (rTMS) targeting the TPJ currently stands as the best-established neuromodulation intervention for treatment-resistant auditory hallucinations (TR-AH). Across multiple randomized controlled trials and independent meta-analyses, the pooled standardized mean difference versus sham is approximately 0.54, corresponding to a number-needed-to-treat of about five, which is clinically meaningful for a highly refractory population. These benefits typically emerge within 2 weeks of treatment and, in the absence of maintenance sessions, persist for a median of 6–8 weeks. Thus, rTMS offers a valuable additional option for patients who do not respond adequately to antipsychotics. However, the need for neuronavigation-compatible equipment, trained technicians, and daily clinic-based delivery restricts rTMS largely to tertiary centers and limits its scalability, particularly in low-resource settings.

Transcranial direct current stimulation (tDCS), most commonly using a cathode over the left TPJ and anode over the left dorsolateral prefrontal cortex (DLPFC), presents a compelling complementary alternative. Although its short-term effect size appears modestly smaller than that of rTMS, tDCS offers several distinctive advantages: an excellent safety profile with a near-zero serious adverse-event rate, minimal side effects, negligible per-session cost, and battery-operated portability that makes supervised home-based administration realistically achievable. For the roughly 80% of individuals with schizophrenia living in low- and middle-income countries, tDCS is currently the only neuromodulatory modality with genuine potential for wide-scale implementation without major capital investment. Nonetheless, the clinical evidence base remains relatively limited, with small sample sizes, heterogeneous protocols, and short follow-up. Large-scale effectiveness trials embedded in routine mental-health services are now urgently needed to confirm preliminary efficacy signals, optimize stimulation parameters and maintenance schedules, and establish remote-supervision protocols that ensure safety, adherence, and treatment fidelity.

Deep brain stimulation (DBS) remains an innovative yet highly experimental option reserved for the most severe, intractable TR-AH cases. The mechanistic rationale—high-frequency modulation of cortico-striato-thalamo-cortical loops to suppress aberrant TPJ activity—is conceptually attractive and aligns with network-based models of psychosis. However, the empirical foundation is extremely limited, comprising fewer than 10 published cases and no randomized evidence. Given its invasiveness, surgical and neuropsychiatric risks, cost, and ethical complexities (including issues of consent capacity, long-term device management, and patient expectations), DBS cannot be considered a routine therapeutic option at present and should be confined to rigorously monitored research protocols.

Across all modalities, several critical cross-cutting barriers impede progress. First, the lack of reliable response predictors means that neuromodulation is typically applied in a “one-size-fits-all” fashion, leading to relatively high non-response and relapse rates. Second, the limited number of well-powered clinical trials and substantial protocol heterogeneity constrain our ability to standardize dosing, target selection, and maintenance strategies. Third, long-term sustainability is poorly understood: relapse trajectories, optimal retreatment intervals, and the durability of functional improvements remain under-characterized. Addressing these gaps will require large multi-center effectiveness RCTs that incorporate longer follow-up, standardized outcome measures, and cross-site harmonization of protocols. A concerted effort is also needed to discover and validate neuroimaging and electrophysiological biomarkers—potentially drawing on emerging work such as Xie et al. (2025) on brain complexity—to stratify patients, guide target selection, and monitor treatment response in real time.

Finally, more attention should be paid to treatment acceptability and integration with psychosocial care. Combining neuromodulation with cognitive-behavioral interventions for voices may enhance both symptom reduction and coping, and could help address concerns about “machine-only” treatments. Developing scalable delivery models—particularly those exploiting the portability and home-use potential of tDCS—will be essential for extending neuromodulation beyond specialized academic centers and embedding it within stepped-care frameworks in routine psychosis services.

### 10.1 Limitations

Several limitations of the existing neuromodulation literature warrant consideration. First, most trials report only mean group-level effects and do not systematically contrast responders with non-responders, thereby limiting the development of individualized predictive models to inform clinical decision-making. Second, long-term outcomes remain insufficiently characterized, as few studies include follow-up periods extending beyond 6–12 months, leaving relapse trajectories and the necessity of maintenance sessions unclear. Third, although neuromodulation is frequently administered alongside antipsychotic treatment, randomized head-to-head comparisons of combined versus monotherapy approaches are largely absent, precluding evidence-based guidance on optimal treatment sequencing. Fourth, dose–response relationships are poorly delineated due to heterogeneous stimulation parameters and the lack of pre-specified dose-based subgroup analyses, which hinders the identification of optimal and cost-effective dosing strategies. Finally, adverse event profiles are derived primarily from randomized trial data and may underestimate rare or delayed harms, particularly as these interventions are translated into broader clinical practice or home-based settings. An additional limitation of this review concerns the pronounced demographic homogeneity of the included studies. The current evidence base is derived almost exclusively from Caucasian populations in Western industrialized countries, substantially limiting the generalizability of the conclusions to more diverse global populations. Addressing this external validity gap will require future research to prioritize inclusive sampling strategies and to conduct systematic cross-cultural validation studies.

### 10.2 Dual diversity crisis: population and neurobiological heterogeneity

Contemporary neuromodulation research for auditory hallucinations faces a “dual diversity crisis” that undermines inference in two linked ways. First, population diversity including demographic, ancestral, and environmental factors is severely underreported and under-sampled (Rudroff, 2026). Most randomized studies originate from Western academic centers with samples collected form western, educated, industrialized, rich, and democratic (WEIRD) population, reducing the evidence base needed to validate models and dose–response relationships across settings (Henrich et al., 2010). Second, neurobiological heterogeneity which is the fact that equivalent symptom scores may arise from distinct circuit dysfunctions (Rudroff, 2025). In AH, temporal-dominant, prefrontal-dominant, and striato-thalamo-cortical patterns all contribute to generation of AH, which means that standardized protocols can mismatch the underlying mechanism. Such issues mean that pooled “average” effects may not apply to many patient subgroups and that high non-response rates could reflect intervention-biology mismatches rather than irreversible treatment resistance.

Mechanistically, several readily measurable factors plausibly modify neuromodulation delivery and effect. Skull thickness and scalp-to-cortex distance that change electric field strength and RMT, cortical folding and regional anatomy that change local field orientation, and genetic differences affecting neurotransmitter function and plasticity should all be incorporated to improve modulation parameters (Vergallito et al., 2022). These inter-individual and population differences have been documented in the neuromodulation literature as sources of variability in tDCS/rTMS outcomes and should be explicitly addressed in future trial designs. Practically, addressing this crisis requires mandatory reporting of participant ancestry/ethnicity and head/anatomical metrics in all trials, inclusion of population-diverse, multi-site cohorts and pre-planned subgroup analyses, use of individualized electric-field modeling when titrating dose, and biomarker-led stratification to match stimulation target and type to the patient’s dominant pathophysiology. These actions will move the field from “one-size-fits-all” protocols to mechanism-matched interventions, strengthening both equity and scientific validity.

## 11 Conclusion

In summary, low-frequency rTMS of the left TPJ currently represents the reference-standard neuromodulation technique for TR-AH, with moderate, time-limited efficacy supported by multiple RCTs and meta-analyses and an acceptable safety profile. tDCS, while supported by a smaller and more heterogeneous evidence base, emerges as a highly pragmatic, low-cost, and scalable alternative with exceptional safety and realistic potential for supervised home-based use, particularly relevant for low- and middle-income settings. DBS remains a mechanistically intriguing but experimental approach that should, for now, be restricted to carefully controlled research studies. Future work should prioritize robust effectiveness trials, biomarker development, combination strategies with psychosocial therapies, and innovative delivery models to enhance personalization, scalability, and long-term sustainability of neuromodulation for TR-AH.
